# Supplementary material for: Circularly polarized light scattering imaging of a cancerous layer creeping under a healthy layer for the diagnosis of early-stage cervical cancer
Source: J Biomed Opt. 2026 Feb 6;31(2):027002. doi: 10.1117/1.JBO.31.2.027002 (PMC12880821; doi:10.1117/1.JBO.31.2.027002)
Supplement: Supplementary file 1 [file JBO_031_027002_SD001.pdf]

Supporting Information

Circularly polarized light scattering imaging for squamous intra-epithelial dysplasia

Nozomi Nishizawa, Mahiro Ishikawa, Mike Raj Maskey, Asato Esumi, Toshihide Matsumoto, Takahiro Kuchimaru

Supplementary Data 1: Intensity and DOCP values in the layered structure

Figure S2 shows the raw images and the DOCP distribution images captured with the polarization imaging system for samples with different T_2 and a fixed T_1 of 0.5 mm.

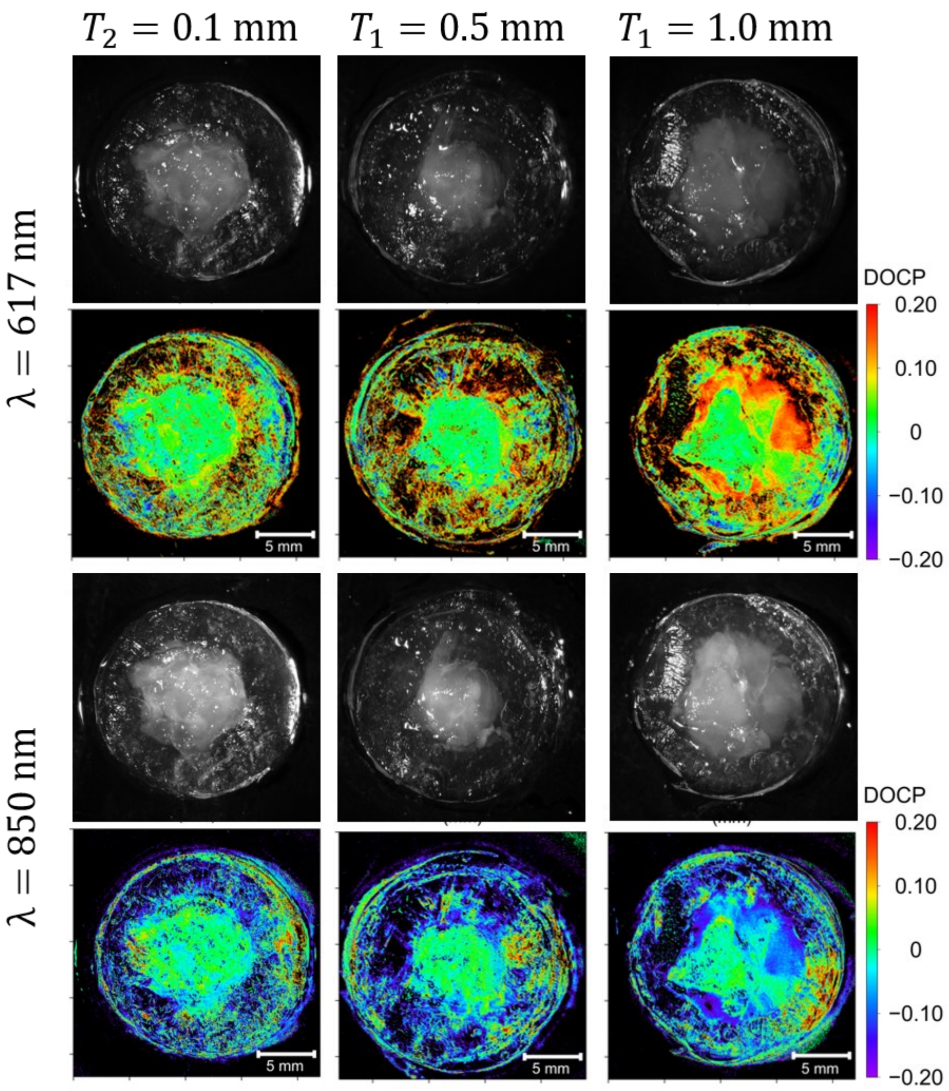

FIGURE S1 Raw images and DOCP distribution images captured with the polarization imaging system for the samples with different T_2 and a fixed T_1 of 0.5 mm. The images captured with wavelengths of 617 nm and 850 nm are in the upper and lower half-rows, respectively, and those for the samples with T_2 of 0.1, 0.5, and 1.0 mm are arranged sequentially from the left.
